# Clinical Outcomes of Oral Traumatic Fibroma Removal Using a 980 nm Diode Laser: A Series of Four Cases

**DOI:** 10.7759/cureus.80390

**Published:** 2025-03-11

**Authors:** Salvatore L La Terra, Gianluigi Caccianiga, Francesco Buoncristiani, Faisal Alzahrani, Faris M Alabeedi

**Affiliations:** 1 Department of Regenerative Cellular Therapy, Faculty of Natural Health Science, Selinus University of Sciences and Literature, London, GBR; 2 Department of Periodontology, Oral Surgery, and Oral Medicine, "La Terra" Private Dental Clinic, Ragusa, ITA; 3 Department of Translational Medicine, University of Ferrara, Ferrara, ITA; 4 Department of Oral Surgery, "Buoncristiani" Private Dental Clinic, San Vincenzo, ITA; 5 Department of Oral and Maxillofacial Surgery, Royal Armed Forces Medical Service, Riyadh, SAU; 6 Department of Oral Surgery, College of Medicine and Dentistry, Ulster University, Birmingham, GBR; 7 Department of Maxillofacial Surgery and Diagnostic Sciences, Faculty of Dentistry, Najran University, Najran, SAU

**Keywords:** benign lesion, diode laser surgery, excision, fibroma, traumatic fibroma

## Abstract

This case series evaluates the clinical outcomes of oral fibroma (traumatic fibroma) excision using a 980 nm diode laser in four patients aged between 35 and 78 years (mean age: 54.25 years). The lesions varied from 0.8 to 1.5 cm (mean: 1.025 cm). All patients underwent laser excision with a 980 nm diode laser (2W, continuous wave, contact mode). Postoperative pain, healing time, recurrence, complications, and patient satisfaction were assessed. All cases demonstrated complete fibroma removal, minimal bleeding, uneventful healing within seven to nine days, and no recurrence at six months. Postoperative pain was mild, and patient satisfaction was high. This case series suggests that a 980 nm diode laser is a safe and effective modality for traumatic fibroma removal in the oral cavity.

## Introduction

Oral fibromas, also known as traumatic fibromas or irritation fibromas, are benign, reactive lesions of the oral mucosa. They typically arise as a result of chronic irritation or trauma, such as cheek biting, denture irritation, or sharp teeth [1,2]. These lesions, while non-neoplastic, are a frequent encounter in dental practice, significantly impacting patient comfort and oral function. Histologically, they are characterized by dense, collagenous connective tissue with a variable degree of epithelial covering [3]. The prevalence was reported to be around 69%, as they are among the most common benign soft tissue lesions in the oral cavity [4]. This high prevalence necessitates efficient and patient-friendly treatment modalities.

Conventional treatment involves surgical excision using a scalpel. While effective in removing the lesion, this method often requires sutures, which can lead to postoperative discomfort, bleeding, and potential complications such as infection or delayed healing [5]. Diode lasers have emerged as a promising alternative in oral surgical procedures. It gained popularity due to its inherent advantages, such as precise tissue ablation and minimized collateral damage to surrounding healthy tissue [6]. The coagulative properties of diode lasers significantly reduce intraoperative bleeding, enhancing visibility and surgical precision. Furthermore, patients undergoing laser excision often experience reduced postoperative pain and accelerated healing, contributing to a more comfortable recovery. The 980 nm diode laser, in particular, has demonstrated effective soft tissue cutting and coagulation capabilities, rendering it highly suitable for procedures such as traumatic fibroma removal in the oral cavity [6].

While the existing body of literature supports the application of diode lasers in various oral surgical procedures, there remains a need for more focused studies specifically addressing oral fibroma removal. This case series aims to contribute to this knowledge gap by assessing the feasibility and clinical outcomes of utilizing a 980 nm diode laser for traumatic fibroma excision in four cases within a clinical setting. Specifically, it seeks to evaluate the efficacy, safety, and patient satisfaction associated with this minimally invasive treatment modality. By documenting the outcomes of these cases, this study aims to provide valuable insights into the potential benefits of diode laser technology in the management of traumatic fibromas in the oral cavity.

## Case presentation

Materials and methods

Study Design and Patient Selection

This prospective case series included four patients diagnosed with clinically evident oral fibromas who underwent 980 nm diode laser treatment from January 2023 to September 2023 in a private clinical setting in Southern Italy. Diagnosis was done through clinical examination and confirmed through histopathological analysis of the excised tissue.

Inclusion Criteria

Patients with clinically diagnosed traumatic fibromas with no systemic diseases affecting healing and age ≥ 18 years, with no restrictions of gender, ethnicity, or socioeconomic status.

Exclusion Criteria

Patients with multiple lesions due to systemic conditions (e.g., neurofibromatosis) or those with insufficient follow-up data, suspected malignancy, or immunocompromised subjects. Patients who did not sign the consent form for treatment and publication.

Procedure and Ethics/Consent

Preoperative assessment included patient history and clinical examination. Following the explanation of the management of the lesions and different therapeutic options, informed consent forms for treatment and publication were signed by all participants. Before the surgery, oral antisepsis was carried out by using a 0.2% chlorhexidine solution. For the surgical excisions, a 980 nm diode laser (Litemedics® Prime, Milan, Italy) with a 320 µm fiber tip, 2 W output power, and continuous wave (CW) contact mode was used under a small amount of local anesthesia (3% mepivacaine). The excision was performed with minimal tissue charring. All treatments were performed by the same specialist without a control group. Postoperative care included oral hygiene instructions, using 0.12% chlorhexidine mouthwash, and taking analgesics (ibuprofen 400-600 mg every four to six hours) as needed. Antibiotics were not prescribed.

Follow-Up and Evaluation of the Outcomes Measured

Postoperative follow-up and evaluation were conducted through scheduled recall visits at three, seven, and 14 days, as well as at one, three, and six months postoperatively. Clinical parameters were systematically assessed to evaluate treatment outcomes. Postoperative pain was quantified using a visual analog scale (VAS) ranging from 0 to 10, with measurements taken at 24 hours and three days following the procedure. Healing time was assessed through the evaluation of mucosal integrity at each follow-up visit. The recurrence rate of the fibroma was monitored at one, three, and six months post treatment. Any complications, such as infection, delayed healing, or scarring, that occurred were planned to be documented. Patient satisfaction was evaluated using a five-point Likert scale, and aesthetic and functional outcomes were also assessed to provide a comprehensive evaluation of the treatment's success.

Data Collection

Patient demographic data, lesion location, size, duration, treatment modalities (surgical excision via diode laser), and histopathological features of the lesions were recorded. Follow-up data regarding recurrence and healing were also collected, as listed in Table 1.

**Table 1:** Summary of clinical features of patients included in the report. NAD: nothing abnormal detected.

Case	Age/sex/medical history	Anatomical location	Clinical features	Clinical diagnosis	Treatment	Histology	Follow-up (months)
1	35/M/NAD	Buccal alveolar mucosa, lower right canine	Appearance: smooth, pink-colored, non-ulcerated sessile nodule, asymptomatic, firm, and resilient. Size: 1.5 cm. Onset: 1 year ago	Traumatic fibroma	Laser-assisted surgical excision (980 nm diode laser)	Nodular mass with dense avascular fibrous stroma & atrophic epithelium	3, 7, and 14 days, 1, 3, and 6 months with no recurrence
2	54/F/NAD	Lower left lip	Appearance: smooth, pink-colored, non-ulcerated sessile nodule, asymptomatic, firm, and rubbery. Size: 0.8 cm. Onset: 2 years ago	Traumatic fibroma	Laser-assisted surgical excision (980 nm diode laser)	Nodular mass with dense avascular fibrous stroma & atrophic epithelium	3, 7, and 14 days, 1, 3, and 6 months with no recurrence
3	78/M/NAD	Right lower lip	Appearance: smooth, pink-colored, non-ulcerated sessile nodule, asymptomatic, firm, and resilient. Size: 1 cm. Onset: 1 year ago	Traumatic fibroma	Laser-assisted surgical excision (980 nm diode laser)	Nodular mass with dense avascular fibrous stroma & atrophic epithelium	3, 7, and 14 days, 1, 3, and 6 months with no recurrence
4	50/M/NAD	Left lower lip	Appearance: smooth, pink-colored, non-ulcerated sessile nodule, asymptomatic, firm, and resilient. Size: 0.8 cm. Onset: 1 year ago	Traumatic fibroma	Laser-assisted surgical excision (980 nm diode laser)	Nodular mass with dense avascular fibrous stroma & atrophic epithelium	3, 7, and 14 days, 1, 3, and 6 months with no recurrence

Results

Summary of Findings

This case series included a total of four patients. The age range of the patients in the examined reports was from 35 to 78 years (mean age: 54.25 years), including three male and one female patients. The anatomical locations of the treated fibromas included lower buccal alveolar mucosa (25%) and labial mucosa of the lower right and left lip, respectively (75%). The maximum diameter of the fibromas ranged from 0.8 to 1.5 cm, with a mean diameter of 1.025 cm. All patients reported a gradual onset of the lesions, spanning from one to two years, with a mean duration of 1.25 years. Each patient presented with a single lesion, simplifying the treatment protocol.

Surgical intervention was performed using a 980 nm diode laser at a power setting of 2.0 W in continuous mode, under local anesthesia (3% mepivacaine). The procedure duration ranged from five to seven minutes, with a mean of 5.75 minutes, indicating the efficiency of the laser treatment. Minimal to no bleeding was observed during and after the procedure, and no sutures were required, contributing to a less invasive approach.

Postoperative healing was uneventful in all cases, with complete mucosal healing observed within seven to nine days, averaging eight days. Absence of recurrence was detected at one-month, three-month, and six-month follow-ups. Postoperative pain was mild (mean VAS score was 2.075 at 24 hours and 1.2 at three days postoperatively) and managed with analgesics. All patients reported high satisfaction with the functional and aesthetic outcomes (mean five-point Likert scale value of 4.5). No complications, such as infection, delayed healing, or scarring, were reported in any of the cases. The histopathological findings in all cases confirmed the clinical diagnosis of traumatic fibroma, characterized by a nodular mass with dense avascular fibrous stroma and atrophic epithelium. A summary of the findings of this study is listed in Tables 2, 3.

**Table 2:** Summary of findings of the study.

Parameter	Findings
Number of cases	4
Age range	35-78 years (mean age: 54.25 years)
Gender distribution	3 males and 1 female
Fibroma location	Lower buccal alveolar mucosa (25%), lower labial mucosa (75%)
Maximum diameter of the lesion	0.8 to 1.5 cm (mean: 1.025 cm)
Onset	1-2 years (mean: 1.25 years)
Diode laser wavelength	980 nm
Power setting	2.0 W continuous mode
Anesthesia used	Local (3% mepivacaine), minimal
Procedure duration	5-7 minutes per lesion (mean: 5.75 minutes)
Bleeding	Minimal; no sutures required
Healing time	7-9 days (mean: 8 days)
Postoperative pain	Mild, managed with over-the-counter analgesics. Mean visual analog scale (VAS) score = 2.075 at 24 hours, 1.2 at 3 days post operation
Recurrence rate	0% (no recurrence at 6-month follow-up)
Complications	None reported
Patient satisfaction	High (all patients satisfied with outcomes. Mean 5-point Likert scale value = 4.5)

**Table 3:** Detailed summary of results for each of the four included cases. OTC: over-the-counter; VAS: visual analog scale.

Parameter	Case 1	Case 2	Case 3	Case 4
Age	35	54	78	50
Gender	Male	Female	Male	Male
Fibroma location	Lower buccal alveolar mucosa	Lower left labial mucosa	Lower right labial mucosa	Lower left labial mucosa
Diode laser wavelength	980 nm	980 nm	980 nm	980 nm
Power setting	2.0 W, continuous mode	2.0 W, continuous mode	2.0 W, continuous mode	2.0 W, continuous mode
Anesthesia used	Local (3% mepivacaine), minimal	Local (3% mepivacaine), minimal	Local (3% mepivacaine), minimal	Local (3% mepivacaine), minimal
Procedure duration	5 minutes	6 minutes	5 minutes	7 minutes
Bleeding	Minimal, no sutures	Minimal, no sutures	Minimal, no sutures	Minimal, no sutures
Healing time	8 days	7 days	9 days	8 days
Postoperative pain	Mild, managed with OTC meds. VAS score 2 at 24 hours and 1.2 at 3 days post operation	Mild, managed with OTC meds. VAS score 1.8 at 24 hours and 1.1 at 3 days post operation	Mild, managed with OTC meds. VAS score 2.2 at 24 hours and 1.3 at 3 days post operation	Mild, managed with OTC meds. VAS score 2.3 at 24 hours and 1.2 at 3 days post operation
Recurrence rate	0% (no recurrence at 6-month follow-up)	0% (no recurrence at 6-month follow-up)	0% (no recurrence at 6-month follow-up)	0% (no recurrence at 6-month follow-up)
Complications	None reported	None reported	None reported	None reported
Patient satisfaction	High, 5-point Likert scale value = 5	High, 5-point Likert scale value = 5	High, 5-point Likert scale value = 4	High, 5-point Likert scale value = 4

Case descriptions

Case 1

Patient information: A 35-year-old male patient was referred to the office for a consultation and a visit as he complained of a slow-growing, cosmetically undesirable soft tissue swelling on his lower alveolar mucosa. It was present for one year and also interfered with mastication. He reported no significant medical or allergic conditions.

**Figure 1:**
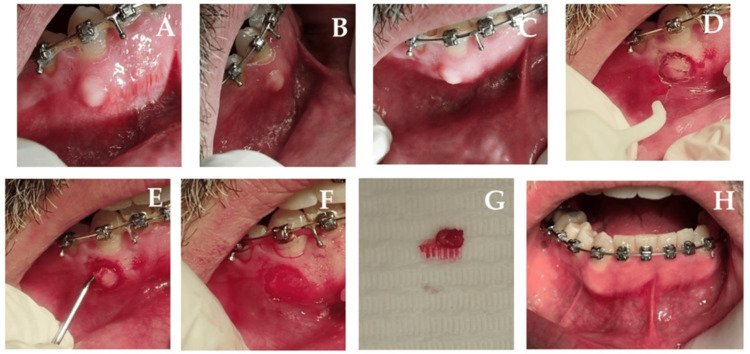
Intraoral clinical view of the lesion at baseline (A-C). Intraoperative view, surgical removal of the lesion via 980 nm diode laser (D-E). Immediate postoperative view (F). Surgical specimen (G). Follow-up at 90 days showing complete healing with no scars or recurrence (H).

Clinical findings: Intraoral examination revealed a smooth, pink, sessile, non-ulcerated, painless, and firm nodule of approximately 1.5 cm (diameter) on the buccal alveolar mucosa adjacent to the lower right canine (Figures 1A-1C). A clinical suspicion of traumatic fibroma was established. Differential diagnoses included pyogenic granuloma, peripheral giant cell granuloma, central giant cell granuloma, and peripheral ossifying fibroma.

Therapeutic intervention: Using minimal local anesthesia (3% mepivacaine), a 980 nm diode laser (320 µm fiber tip, 2 W, continuous wave contact mode) was employed to remove this lesion completely through a circumferential incision. There was no need to suture, and only minimal intra- and postoperative bleeding was noticed. The procedure took overall five minutes to complete (Figures 1D-1G). Postoperative care included oral hygiene instructions and using 0.12% chlorhexidine mouthwash and analgesics (ibuprofen 400-600 mg every four to six hours) as needed. Antibiotics were not prescribed.

Diagnostic focus and assessment: Malignancy and other possibilities were eliminated following the histological analysis. It confirmed a traumatic fibroma, showing a nodular mass of dense avascular fibrous stroma with atrophic epithelium.

Follow-up and outcomes: Follow-up at three, seven, and 14 days, and one, three, and six months revealed complete healing within eight days, with minimal postoperative discomfort (VAS score of 2 at 24 hours and 1.2 at three days) and no recurrence at six months. The patient reported high satisfaction (five-point Likert scale score = 5). Figure 1H shows the clinical view at 90 days.

Case 2

Patient information: A 54-year-old female patient presented to our facility with a two-year history of a gradually developing, painless, and cosmetically undesirable round mass on her lower left lip. She had no significant medical history or allergies.

**Figure 2:**
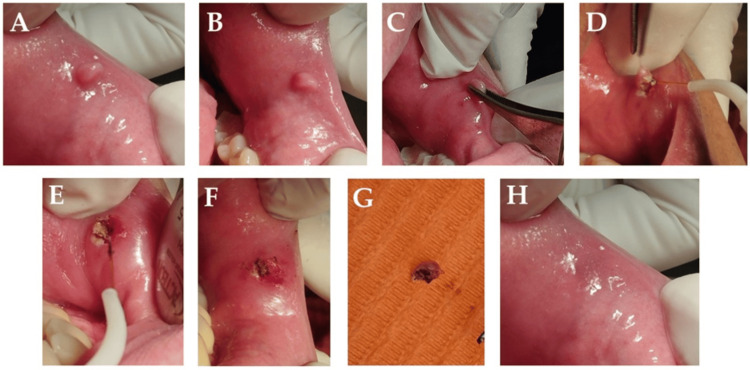
Intraoral clinical view of the lesion at baseline (A-B). Intraoperative view, surgical removal of the lesion via 980 nm diode laser (C-E). Immediate postoperative view (F). Excised surgical specimen (G). Follow-up at 90 days (H) showing complete healing without scars or reoccurrence.

Clinical findings: Intraoral examination revealed a 0.8 cm, smooth, pink, non-ulcerated, sessile nodule on the lower left labial mucosa, which was painless, firm, and rubbery (Figures 2A, 2B). A clinical diagnosis of traumatic fibroma was suspected, with differential considerations including pyogenic granuloma, peripheral and central giant cell granulomas, and peripheral ossifying fibroma.

Therapeutic intervention: Using a small amount of 3% mepivacaine, a 980 nm diode laser (320 µm fiber tip, 2 W, continuous wave contact mode) was used to perform a complete circumferential excision. No sutures were necessary, and minimal bleeding was observed during and after the six-minute procedure (Figures 2C-2G). Postoperatively, oral hygiene instructions were provided, and 0.12% chlorhexidine mouthwash and analgesics (ibuprofen 400-600 mg every four to six hours) were prescribed. Antibiotics were not used.

Diagnostic focus and assessment: Histopathological analysis confirmed the clinical diagnosis. It revealed a nodular mass of dense collagen bundles with fibroblasts and atrophic rete ridges present in the overlying stratified squamous epithelium.

Follow-up and outcomes: Complete tissue healing without complications was observed by the seventh day, with minimal postoperative discomfort (VAS score of 1.8 at 24 hours and 1.1 at three days) and no recurrence at six months. Follow-ups were at three, seven, and 14 days, and one, three, and six months. The patient reported high satisfaction (five-point Likert scale score = 5) (Figure 2H).

Case 3

Patient information: Our clinic attended to a 78-year-old male presenting with a one-year history of a soft tissue lesion on his lower right labial mucosa. His medical history was negative for systemic diseases and allergies.

**Figure 3:**
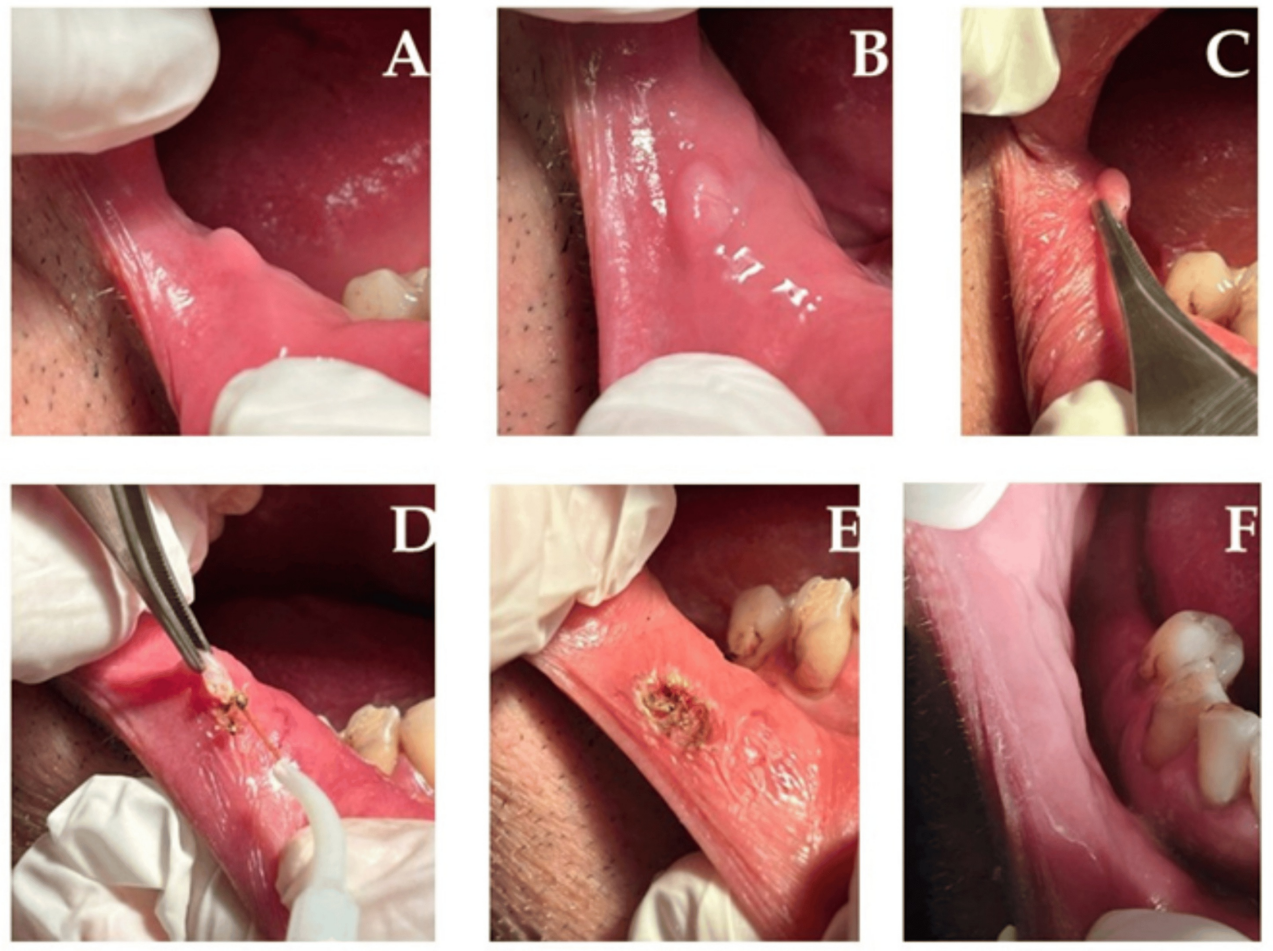
Intraoral clinical view of the lesion at baseline (A-B). Intraoperative view, surgical removal of the lesion via 980 nm diode laser (C-D). Immediate postoperative view (E). Follow-up at 90 days (F) showing complete healing without scars or reoccurrence.

Clinical findings: Intraoral examination revealed a 1 cm, smooth, pink, non-ulcerated, sessile, firm nodule (Figures 3A, 3B). Clinical evaluation led to a suspected diagnosis of traumatic fibroma, with differential considerations including pyogenic granuloma, peripheral and central giant cell granulomas, and peripheral ossifying fibroma.

Therapeutic intervention: A minimal amount of 3% mepivacaine was used for local anesthesia, and a 980 nm diode laser (320 µm fiber tip and 2 W output power, continuous wave mode in contact mode) was used. The procedure was completed in five minutes, with minimal bleeding and no need for sutures (Figures 3C-3E). After surgery, oral hygiene instructions were provided, and 0.12% chlorhexidine mouthwash and analgesics (ibuprofen 400-600 mg every four to six hours) as required were prescribed. Antibiotics were not used.

Diagnostic focus and assessment: Histopathological analysis confirmed a traumatic fibroma, revealing a dense avascular fibrous stroma with atrophic epithelium.

Follow-up and outcomes: Follow-up appointments at three, seven, and 14 days, and one, three, and six months demonstrated tissue healing within nine days, with an uneventful postoperative recovery (VAS score of 2.2 at 24 hours and 1.3 at three days). Clinical review showed complete healing, normal gingival color, and absence of recurrence. The patient reported positive functional and aesthetic outcomes (five-point Likert scale score = 4) (Figure 3F).

Case 4

Case presentation: A 50-year-old male patient presented to our facility with a one-year history of a gradually developing, painless, and cosmetically undesirable round mass on his lower left labial mucosa. He had no significant medical history or allergies.

**Figure 4:**
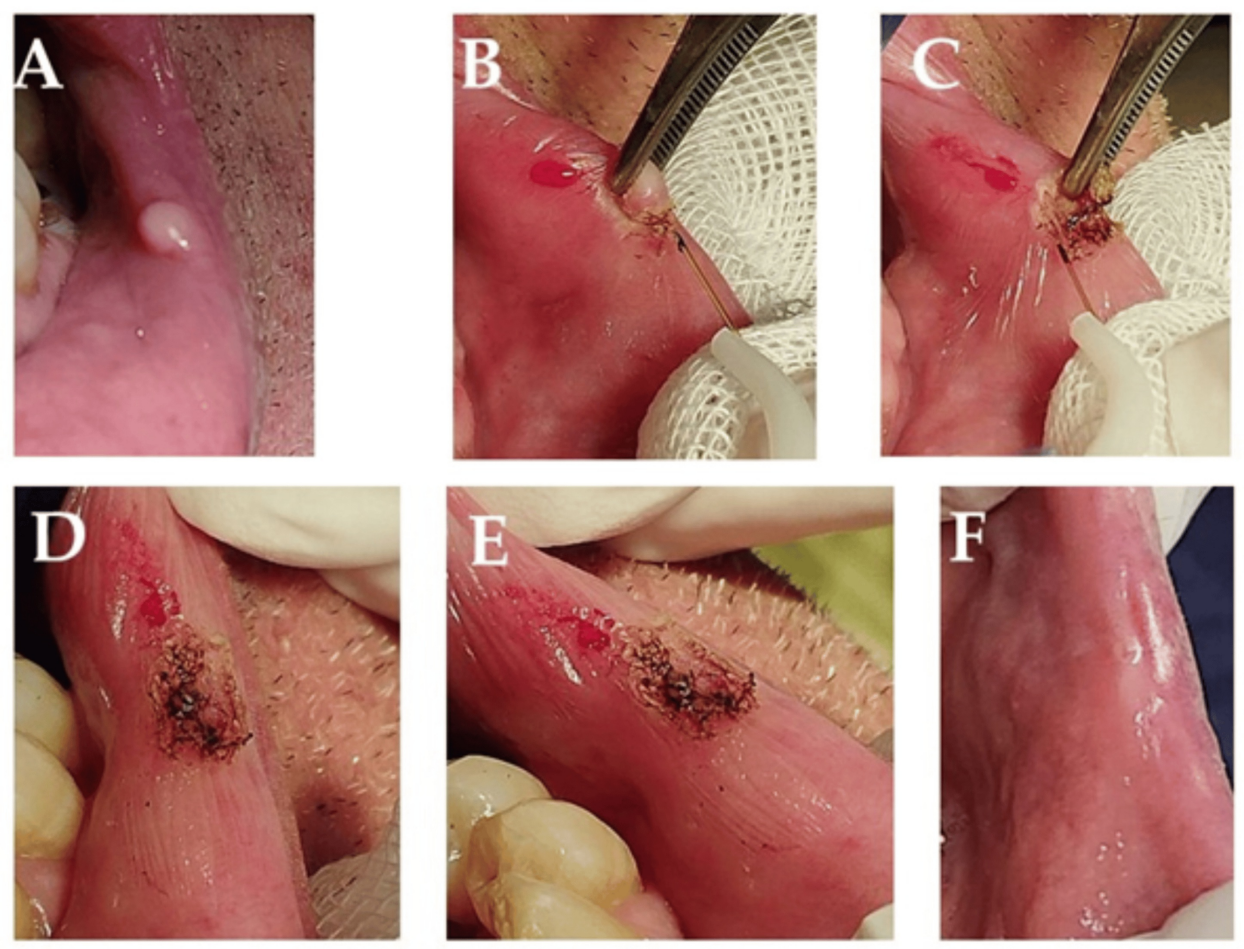
Intraoral clinical view of the lesion at baseline (A). Intraoperative view, surgical removal of the lesion via 980 nm diode laser (B-C). Immediate postoperative view (D-E). Follow-up at 90 days (F) showing complete healing without scars or reoccurrence.

Clinical findings: Intraoral examination revealed a 0.8 cm smooth, pink, non-ulcerated, sessile nodule on the lower left labial mucosa, which was painless, firm, and rubbery (Figure 4A). A clinical diagnosis of traumatic fibroma was suspected, with differential considerations including pyogenic granuloma, peripheral and central giant cell granulomas, and peripheral ossifying fibroma.

Therapeutic intervention: Using a small amount of 3% mepivacaine, a 980 nm diode laser (320 µm fiber tip, 2 W, continuous wave contact mode) was used to perform a complete circumferential excision. No sutures were necessary, and minimal bleeding was observed during and after the seven-minute procedure (Figures 4B-4E). Postoperative care included oral hygiene instructions and using 0.12% chlorhexidine mouthwash; analgesics (ibuprofen 400-600 mg every four to six hours) were prescribed as required, and antibiotics were withheld.

Diagnostic focus and assessment: Histopathological analysis confirmed the clinical diagnosis, revealing a nodular mass of dense collagen bundles with fibroblasts and atrophic rete ridges.

Follow-up and outcomes: Complete tissue healing without complications was observed by the eighth day, with minimal postoperative discomfort (VAS score of 2.3 at 24 hours and 1.2 at three days) and no recurrence at six months. Follow-ups were done at three, seven, and 14 days, and one, three, and six months. The patient reported high satisfaction (five-point Likert scale score = 4) (Figure 4F).

## Discussion

This case series demonstrates the successful application of a 980 nm diode laser for the excision of traumatic fibromas, highlighting its efficacy and safety in a clinical setting. The use of diode lasers in soft-tissue procedures offers distinct advantages over traditional scalpel excision [7,8]. Their affinity for melanin and hemoglobin allows for precise cutting and simultaneous coagulation, resulting in minimal bleeding, enhanced visibility, and improved patient comfort. This minimally invasive approach reduces tissue trauma, accelerates healing, and yields better cosmetic outcomes [8].

The findings of this case series align with published reports with established benefits of diode lasers [9], as evidenced by reduced postoperative pain (mean VAS scores of 2.075 at 24 hours and 1.2 at three days), rapid healing (seven to nine days), and minimal intraoperative and postoperative bleeding [10]. These outcomes are consistent with previous studies demonstrating the hemostatic capabilities and reduced inflammatory response of diode lasers compared to scalpel surgery [11-13]. The minimal bleeding observed is attributed to the coagulative effects of the diode laser [11,14]. This mechanism effectively seals blood vessels during tissue removal. Considerably lower discomfort may be attributed to the lower inflammatory response induced by laser energy compared to scalpel surgery [15]. The absence of sutures, contributing to natural mucosal regeneration, further explains the accelerated healing observed [16]. While a diode laser alone proves effective, a combination with a scalpel may be considered for large fibromas near the retromolar pad region [5,8,11].

The successful removal of fibromas, with no recurrence at six months, reinforces the efficacy of laser ablation in oral soft tissue surgery [13,17]. The efficiency of the procedure is further supported by its short duration (five to seven minutes) and minimal complications, making it feasible for routine clinical practice. In a recent case series, diode lasers using 810 to 980 nm were used for excision of irritational fibroma in three female patients, aged nine, 39, and 45 years [13]. Nevertheless, it is important to use the correct protocol while working on diode lasers [18]. The primary advantages observed were minimal bleeding, reduced postoperative pain, rapid healing, and high patient satisfaction. These factors collectively contribute to a more comfortable and efficient treatment experience.

Limitations and future research suggestions

This case series has limitations. The small sample size (n = 4) limits the generalizability of the findings. The absence of a control group precludes a direct comparison with traditional scalpel excision. Moreover, the relatively short follow-up period of six months may not capture long-term recurrence rates. Hence, the results may not apply to more extensive or recurrent fibromas. Future research should include larger sample sizes, a control group, longer follow-up periods, and multicenter studies to validate these findings. A larger study would also help to evaluate the efficacy.

Implications for practice

Clinically, the 980 nm diode laser provides a valuable alternative to traditional excision. This is particularly significant in sensitive areas where precision and minimal bleeding are crucial. This approach prioritizes patient comfort and rapid healing.

In case 4, minimal carbonization was observed due to the laser's interaction with a small bleeding vessel. The 980 nm wavelength's high absorption by hemoglobin leads to coagulation and potential charring, particularly in vascular areas. This highlights the importance of precise laser application and careful management of vascular sites [19].

## Conclusions

The 980 nm diode laser was found to be an effective and promising minimally invasive option for the surgical removal of oral fibroma lesions in patients aged between 35 and 78 years and lesions averaging 1.025 cm in diameter. Patients can have significant benefits, including healing within seven to nine days, minimal discomfort, the absence of sutures, reduced intraoperative bleeding, and pleasing cosmetic outcomes. To validate these findings and assess long-term efficacy, further studies involving larger patient populations and extended follow-up periods are warranted.
